# Compiler-aided systematic construction of large-scale DNA strand displacement circuits using unpurified components

**DOI:** 10.1038/ncomms14373

**Published:** 2017-02-23

**Authors:** Anupama J. Thubagere, Chris Thachuk, Joseph Berleant, Robert F. Johnson, Diana A. Ardelean, Kevin M. Cherry, Lulu Qian

**Affiliations:** 1 Bioengineering, California Institute of Technology, 1200 East California Boulevard, Pasadena, California 91125, USA; 2 Computer Science, California Institute of Technology, 1200 East California Boulevard, Pasadena, California 91125, USA; 3 Applied and Computational Mathematics, California Institute of Technology, 1200 East California Boulevard, Pasadena, California 91125, USA

## Abstract

Biochemical circuits made of rationally designed DNA molecules are proofs of concept for embedding control within complex molecular environments. They hold promise for transforming the current technologies in chemistry, biology, medicine and material science by introducing programmable and responsive behaviour to diverse molecular systems. As the transformative power of a technology depends on its accessibility, two main challenges are an automated design process and simple experimental procedures. Here we demonstrate the use of circuit design software, combined with the use of unpurified strands and simplified experimental procedures, for creating a complex DNA strand displacement circuit that consists of 78 distinct species. We develop a systematic procedure for overcoming the challenges involved in using unpurified DNA strands. We also develop a model that takes synthesis errors into consideration and semi-quantitatively reproduces the experimental data. Our methods now enable even novice researchers to successfully design and construct complex DNA strand displacement circuits.

## Abstract (web summary)

DNA circuits hold promise for advancing information-based molecular technologies, yet it is challenging to design and construct them in practice. Thubagere *et al*. build DNA strand displacement circuits using unpurified strands whose sequences are automatically generated from a user-friendly compiler.

The success of computer engineering has inspired attempts to use hierarchical and systematic approaches for developing molecular devices with increasing complexity. To enable the design and construction of a wide range of functional molecular systems, we need software tools such as a compiler that can automatically translate high-level functions to low-level molecular implementations and provide models and simulations for predicting and debugging the behaviours of designed molecular systems. The mechanism of DNA strand displacement has been used to create a variety of synthetic molecular systems including circuits, motors and triggered assembly of structures^1^. Software tools have been developed for designing and analysing DNA strand displacement systems, capable of generating nucleic acid sequences from well-defined structures and molecular interactions^2^,^3^, calculating the thermodynamic^2^,^4^,^5^ and kinetic^6^ properties of designed molecules, and evaluating if the behaviours of the molecular systems agree with the higher-level designs^3^,^7^,^8^,^9^,^10^,^11^. There also exist a few molecular compilers that can translate abstract functions such as a logic function to DNA strand displacement implementations without requiring an understanding of the molecular level details^12^,^13^. However, there has been little independent experimental validation of these compilers, most of which were developed in parallel with or after experimental findings^12^,^14^.

In addition to software tools that facilitate automated design and analysis of DNA strand displacement circuits, we also need to simplify the experimental procedures for creating these circuits *in vitro*, so that it is possible for researchers with diverse backgrounds to build their own circuits and explore potential applications. A great inspiration is DNA origami^15^, a technique that folds DNA into sophisticated structures. In just 10 years since its birth, DNA origami has become one of the most significant successes in the field of DNA nanotechnology. Over 170 research groups have contributed to advancing this technique or developing it for applications in a variety of research areas^16^,^17^,^18^,^19^. A fundamental reason why DNA origami was able to quickly spread around the world is that the experimental procedure is extremely simple and makes use of cheap, unpurified nucleic-acid strands. In contrast, other than a few very simple circuits with just one or two double-stranded components^20^, most DNA strand displacement circuits were constructed using strands that were purchased either purified or unpurified, but all followed by in-house polyacrylamide gel electrophoresis (PAGE) purification to reduce undesired products due to synthesis errors and stoichiometry errors^12^,^14^,^21^. Purified strands are approximately ten times more expensive than unpurified strands, which significantly increases the cost for building large-scale DNA circuits. In-house PAGE purification is both time consuming and labour intensive.

In this work, we show that one can successfully build a complex DNA strand displacement circuit, using DNA sequences automatically generated from a molecular compiler. We also show that one can even do so using cheap, unpurified DNA strands, following simple and systematic experimental procedures.

## Results

### Circuit design

**Figure 1:**
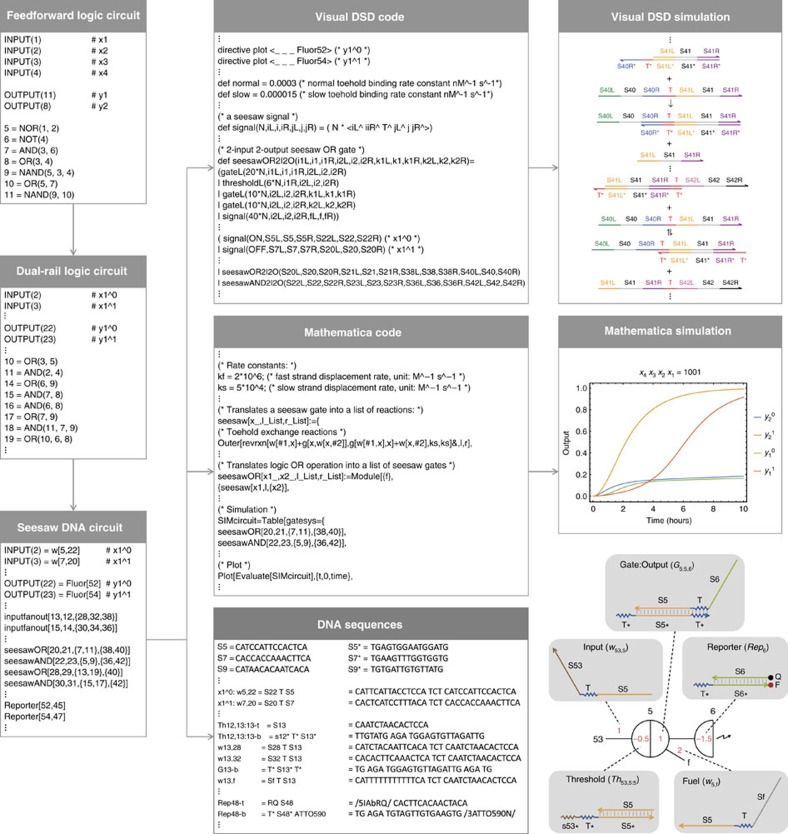
Automated circuit design steps using the Seesaw Compiler. A feedforward digital logic circuit is first translated into an equivalent dual-rail logic circuit and then translated into an equivalent seesaw DNA circuit. Visual DSD code and Mathematica code are generated for analysing and simulating the seesaw DNA circuit, and DNA sequences are generated for constructing the circuit. Bottom right diagram introduces the notations of seesaw circuits: black numbers indicate identities of nodes. The locations and values of red numbers indicate the identities of distinct DNA species and their relative initial concentrations, respectively.

A simple DNA strand displacement motif called the seesaw gate was developed to scale up the complexity of DNA circuits^22^ and was used to demonstrate digital logic computation^12^ and neural network computation^23^. The Seesaw Compiler^12^,^24^ was developed to automatically translate an arbitrary feed forward digital logic circuit into its equivalent seesaw DNA circuit (Fig. 1). The compiler takes an input file that describes a logic circuit with a list of input and output terminals, and a list of AND, OR, NOT, NAND and NOR gates with the connectivity of their terminals specified. First, a technique called dual-rail logic is applied to translate the original logic circuit into an equivalent circuit that contains AND and OR gates only^25^. This is because the NOT gate cannot be directly implemented in multi-layer use-once DNA circuits, if the OFF and ON state of a signal is represented by low and high concentration of a single DNA strand, respectively. If a NOT gate were implemented this way, then output molecules of the gate could be immediately produced in the absence of input. However, once this reaction reaches equilibrium it cannot be reversed, even if input molecules are added at a later point. With dual-rail logic, each terminal in the original circuit is replaced by two terminals, representing the OFF and ON states of a signal separately (for example, each input signal *x*_*i*_ is replaced by 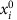 and 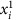). Thus, no reaction will take place until signal molecules on one of the two wires have arrived. With this representation, the NOT gate can be implemented by exchanging the two wires of an input and output signal. Each AND, OR, NAND and NOR gate in the original circuit is replaced by a pair of AND and OR gates.

Next, the compiler translates the dual-rail logic circuit into an equivalent seesaw DNA circuit. In a seesaw DNA circuit, each signal is defined as a wire *w*_*j*,*i*_ connecting seesaw nodes *j* and *i*, and implemented using a single-stranded DNA molecule. Each AND and OR gate in the dual-rail circuit is replaced by a seesaw AND and OR gate, respectively, which is defined as a pair of integrating and amplifying seesaw nodes connected with a set of input and output wires^12^. The seesaw nodes are composed of double-stranded threshold and gate:output molecules and single-stranded fuel molecules (Fig. 1, bottom right). We will explain how the seesaw logic gates work in the next section. Input fan-out gates are introduced to take an input signal that is used for multiple logic gates and produce the corresponding number of output signals. Reporters are introduced to take each output signal and generate a distinct fluorescence signal for readout.

Finally, the compiler generates Visual DSD^3^,^26^ code and Mathematica code for simulating and analysing the seesaw DNA circuit and a file that contains DNA sequences for all molecular species in the circuit. The Visual DSD code can be used to automatically produce diagrams of species, reactions and network graphs with domain-level representation of DNA and to simulate the circuit behaviour based on the network of chemical reactions. The Mathematica code provides more customized and efficient simulations of seesaw circuits. The simulation uses the CRNSimulator package^27^ and models a specific set of side reactions in addition to the designed reactions in a seesaw network^12^.

As a demonstration of using the Seesaw Compiler, we designed a single DNA strand displacement circuit that implements two distinct elementary cellular automata transition functions. An elementary cellular automaton (CA) is one of the simplest models of computation^28^. It consists of a one-dimensional grid of cells, collectively called a generation, where each cell has a binary state of 0 or 1. In each subsequent generation, the state for a cell *C* is determined by its current state and those of its left neighbour *L* and right neighbour *R*. A state transition rule maps each of the 2^3^=8 possible combinations of states for *L*, *C* and *R* to either 0 or 1. Thus, a length 8 binary string uniquely identifies one of the 2^8^ possible transition functions that specify how an elementary CA will evolve between generations. The rule 110 elementary CA (binary number 01101110 written in decimal) is famously known to be Turing universal^29^.

**Figure 2:**
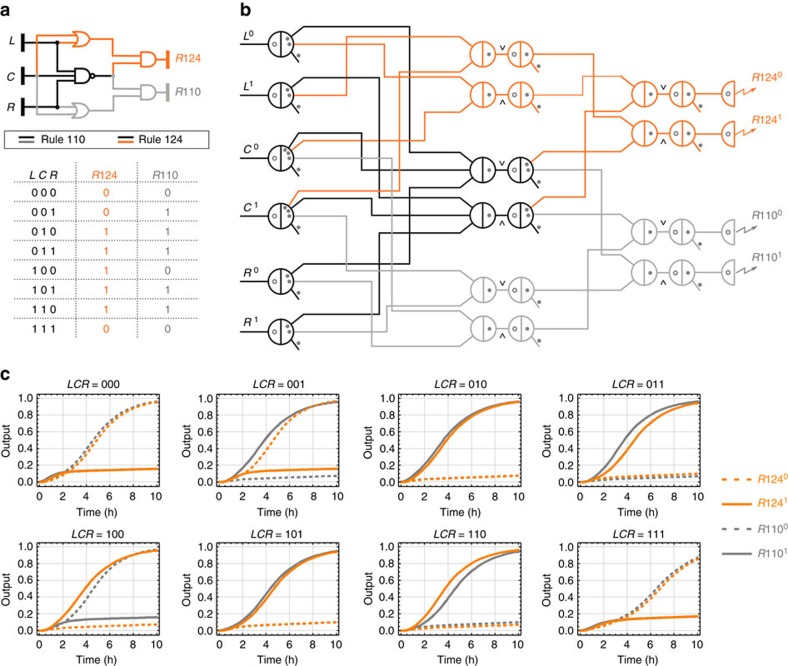
Design of a rule 110–124 circuit using the Seesaw Compiler. (**a**) Gate diagram and truth table of a digital logic circuit that computes the transition rules 110 and 124 of elementary cellular automata. (**b**) Seesaw gate diagram of the equivalent DNA strand displacement circuit. Each seesaw node connected to a dual-rail input implements input fan-out. Each pair of seesaw nodes labelled 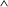 and 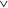 implements a dual-rail AND and OR gate, respectively. Each pair of dual-rail AND and OR gates implements an AND, OR or NAND gate in the original logic circuit. Each dual-rail output is converted to a fluorescence signal through a reporter, indicated as a half node with a zigzag arrow. Each circle and dot inside a seesaw node indicates a double-stranded threshold and gate molecule, respectively. Each dot on a wire indicates a single-stranded fuel molecule. (**c**) Simulations of the DNA strand displacement circuit using the previously developed model for purified seesaw circuits. Trajectories and their corresponding outputs have matching colours. Overlapping trajectories were shifted to be visible. Dotted and solid lines indicate dual-rail outputs that represent logic OFF and ON, respectively. For example, when input *LCR*=001, meaning *L*^0^, *C*^0^ and *R*^1^ were introduced at a high concentration and *L*^1^, *C*^1^ and *R*^0^ at a low concentration, two output trajectories *R*124^0^ and *R*110^1^ reached an ON state and the other two output trajectories *R*124^1^ and *R*110^0^ remained in an OFF state, indicating that the output was computed to be 0 and 1 for rule 124 and 110, respectively. Simulations were performed at 1 × =50 nM—the compiler recommended standard concentration for large-scale purified seesaw circuits.

Another rule that is equally powerful is rule 124 (binary number 01111100 written in decimal), generated by applying the following mirror transformation: the new state of the centre cell for *LCR*=*zyx* in rule 124 is the same as the new state for *LCR*=*xyz* in rule 110. Our circuit was designed to compute a combined logic function of the two transition rules (Fig. 2a). It consists of five logic gates in two layers, including a three-input two-output NAND gate. It is noteworthy that we designed the circuit to demonstrate an interesting logic function associated with cellular automata and not to implement the actual cellular automata model. The circuit operates in a well-mixed test tube environment that does not involve spatial dynamics (that is, no geometry of cells).

The DNA circuit generated by the Seesaw Compiler consisted of 6 layers and a total of 78 distinct initial DNA species (Fig. 2b and Supplementary Fig. 1). Mathematica simulations of the DNA circuit predicted correct computation for all 8 possible input combinations under ideal experimental conditions (Fig. 2c).

The next step was to construct the DNA circuit using strands that were purchased unpurified and with no additional in-house purification. We expected that the main challenges would be to understand how synthesis errors and stoichiometry errors affect the behaviours of DNA circuits and to explore solutions that restore the desired circuit behaviour. We took a bottom-up approach and began building the DNA circuit from the simplest functional component—digital signal restoration.

### Calibrating effective concentrations

Digital signal restoration is a process that pushes the intrinsically analog signal towards either the ideal ON or OFF state, therefore cleaning up the noise and compensating for the signal decay that occurs during circuit execution. In seesaw circuits, digital signal restoration is a component of every logic gate, and is implemented by an amplifying seesaw node with the following idealized input-output function:


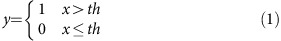


At the molecular level, the digital signal restoration process consists of two basic reactions: catalysis and thresholding. Catalysis is implemented with two toehold exchange pathways that release free output strands *w*_*i*,*k*_ from double-stranded gate molecules *G*_*i*:*i*,*k*_, using the input strands *w*_*j*,*i*_ as a catalyst (Supplementary Fig. 2a):


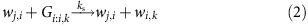


Catalysis can be used for signal amplification, since a small amount of input can trigger the release of a much larger amount of output.

Thresholding is implemented with double-stranded threshold molecules *Th*_*j*,*i*:*i*_ consuming the input at a much faster rate (*k*_f_≫*k*_s_) than the input acting as a catalyst (Supplementary Fig. 2b):


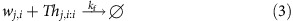


**Figure 3:**
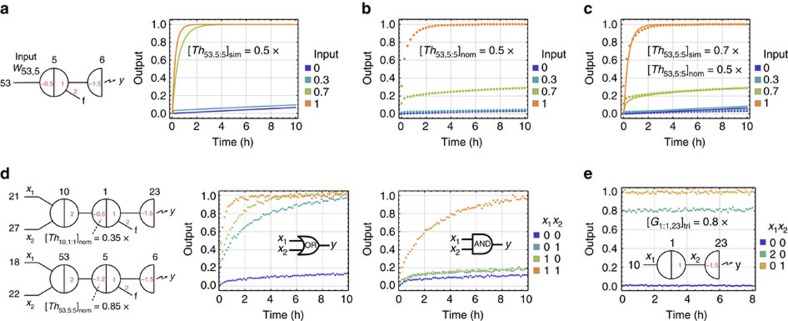
Calibrating effective concentrations. (**a**) Simulations and (**b**) experimental data of digital signal restoration. (**c**) Estimating effective threshold concentration by fitting simulations to the data obtained. (**d**) OR and AND logic gates constructed using adjusted nominal threshold concentrations. (**e**) Estimating effective gate concentration. Data show steady-state fluorescence level. 1 × =100 nM. Here and in later figures, all output signals in the data were normalized using the minimum fluorescence signal (the first data point) of an OFF trajectory as 0 and the maximum fluorescence signal (the average of the last five data points) of an ON trajectory as 1.

As shown in simulations generated using the Seesaw Compiler (Fig. 3a), when the concentration of the threshold molecule is 0.5 × (where 1 × is a standard concentration of 100 nM), we expect that input less than the threshold (for example, 0.3 × ) should be cleaned up to an ideal OFF state via reaction 3 and input greater than the threshold (for example, 0.7 × ) should be amplified to an ideal ON state via reaction 2. However, the observed circuit behaviour was different: when input=0.7 × , the output signal was higher than an ideal OFF state, but did not reach an ideal ON state (Fig. 3b). This experimental result suggested that the input did not sufficiently exceed the threshold, which was an indication that the effective concentration of an unpurified threshold species, compared with that of an unpurified signal species, was higher than expected.

The nominal concentration of a DNA species can be measured using ultraviolet absorbance, but it can be higher than the effective concentration, which is the concentration of the DNA species actually performing the desired reactions. If the sequences of the DNA strands are properly designed, the difference between nominal concentration and effective concentration is typically caused by synthesis errors including nucleotide insertion, deletion and mismatch. To calibrate the effective concentrations of unpurified DNA molecules, we defined the following ratio between effective (eff) and nominal (nom) concentrations of an arbitrary signal, threshold and gate species:


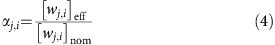



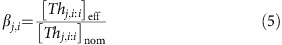



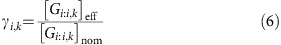


The effective to nominal concentration of a DNA species cannot be measured in isolation. More importantly, the absolute values of *α*, *β* and *γ* should only affect the speed but not the correctness of computation, if the values remain comparable to each other. Thus, we chose to estimate the ratio between *β* and *α* for a threshold consuming a signal, by comparing simulation with experimental result of a signal restoration circuit. For example, manipulating the threshold value in simulation (sim) identified that 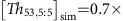 agreed with the experimental data (Fig. 3c), which means the effective concentration of the threshold was similar to that of the signal for 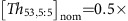 and 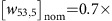. Thus, the threshold to signal ratio can be calculated as:


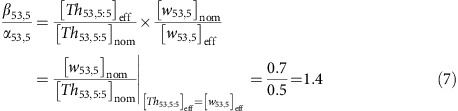


A possible explanation for an unpurified threshold having a higher effective concentration than an unpurified signal, when the nominal concentrations are the same, is the following: the synthesis errors of an unpurified strand depend on the length of the strand, because in the process of chemical synthesis each nucleotide is attached to a growing chain of oligonucleotide one at a time and the coupling efficiency of each step is less than 100% (ref. 30). Threshold molecules are composed of shorter strands (15 and 25 nucleotides) than signal molecules (33 nucleotides) and thus may contain fewer synthesis errors.

Additional signal restoration experiments suggested that the threshold to signal ratio *β*/*α*=1.4 was consistent for different threshold and signal molecules (Supplementary Fig. 3). Thus, using this ratio, we can then calculate how to adjust the nominal thresholds for correctly computing logic AND and OR.

Each seesaw logic gate has an integrating node upstream of an amplifying node. Ideally, an integrating node outputs the sum of all inputs:


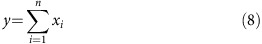


A two-input logic function can be computed as:


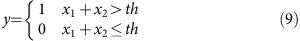


Assuming that an ideal OFF state is [0, 0.2] and an ideal ON state is [0.8, 1], *th*=0.6 will compute logic OR and *th*=1.2 will compute logic AND, if the effective concentrations of the threshold and input signals are comparable to each other (that is, *β*/*α*=1).

As *β*/*α*≠1 for unpurified threshold and signal molecules, we can take this ratio into consideration while calculating the lower and upper bounds of the nominal threshold for an *n*-input logic gate:


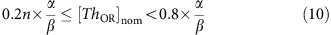



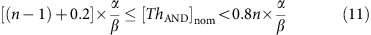


Using *β*/*α*=1.4, we chose a nominal threshold of 0.35 × and 0.85 × for two-input OR and AND gate, respectively, and 0.4 × and 1.6 × for three-input OR and AND gate. Experiments of the logic gates showed desired behaviours (Fig. 3d and Supplementary Fig. 4).

An alternative approach for adjusting the nominal threshold is to use the following equations:


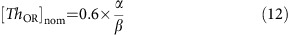



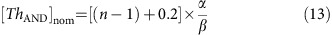


Compared with choosing a nominal threshold based on the lower and upper bounds, this approach is less flexible but simpler.

Next, we can estimate the ratio between *γ* and *α* for a gate releasing a signal, using an experiment that compares the fully triggered (tri) concentration of the gate with the signal when their nominal concentrations are the same. For example, the data in Fig. 3e showed that 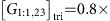 when 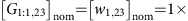. Thus, the gate to signal ratio can be calculated as:


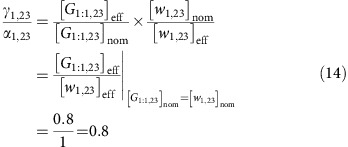


Additional gate calibration experiments suggested that the ratio *γ*/*α*=0.8 was consistent for different gate and signal molecules (Supplementary Fig. 5). We suspect that due to synthesis errors in gate molecules, not all gates can successfully release a signal, which is why an unpurified gate has a lower effective concentration compared to a signal.

As signal restoration was built in within every logic gate to accept an ON state of [0.8, 1], we decided not to make any adjustment for nominal gate concentrations if *γ*/*α*≥0.8. Otherwise, nominal concentration of an amplifying gate and an *n*-input integrating gate can be adjusted as:


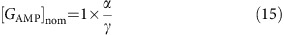



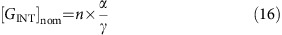


Importantly, the values of *α*, *β* and *γ* should depend on the strand quality and thus could vary with different DNA synthesis providers, procedures and even batches. It is necessary to recalculate the ratios *β*/*α* and *γ*/*α*, if these conditions change.

### Identifying outliers

**Figure 4:**
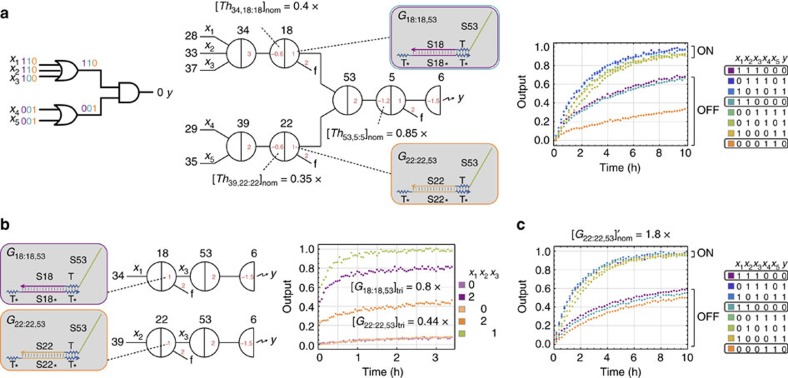
Identifying an outlier gate. (**a**) Logic circuit diagram, seesaw circuit diagram and experimental data of a two-layer logic circuit. (**b**) Measuring the effective concentrations of the gate species. Three independent circuits were used to measure the effective concentrations of two gates fully triggered by *x*_1_ and *x*_2_, respectively, comparing with the effective concentration of *x*_3_ (using signal strand *w*_18,53_). (**c**) Experimental data of the two-layer logic circuit using adjusted nominal gate concentration. 1 × =100 nM.

With calibrated logic gates, we investigated how well they compose together in larger circuits. We constructed a two-layer logic circuit that is part of the rule 124 sub-circuit and is composed of an AND gate and two upstream OR gates (Fig. 4a). The expected circuit behaviour is that the output should remain OFF when only one of the upstream OR gates is ON. However, the observed circuit behaviour showed that the output was reasonably OFF when one upstream OR gate was ON, but was half ON when the other upstream OR gate was ON. This experimental result suggested that the ON signals pushed onto the two input wires of the downstream AND gate (that is, the output wires of the two upstream OR gates) were significantly different from each other, which was an indication that the effective concentrations of the two unpurified gate species that released the output signals were different—one of the gates must be an outlier with *γ*/*α*≠0.8.

Indeed, with a gate calibration experiment shown in Fig. 4b, we measured that *γ*_18,53_/*α*_18,53_=0.8 × for one gate and *γ*_22,53_/*α*_22,53_=0.44 × for another. A possible explanation is that the synthesis errors of unpurified strands somewhat depend on DNA sequences^30^ and variations of effective concentrations may occur between different gate or threshold species. We suspect it was not a coincidence that the outlier gate had a lower effective concentration compared with other unpurified gates, because a particular DNA strand having much worse quality than average is probably more likely than it having much better quality.

Once an outlier is identified, either a threshold or a gate, the nominal concentration can be adjusted using its own threshold to signal ratio (that is, *β*/*α*) or gate to signal ratio (that is, *γ*/*α*), the common nominal concentration described in the previous section, and the common ratio for other thresholds and gates:


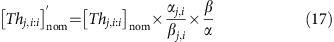



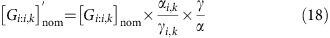


We constructed the two-layer logic circuit using the adjusted nominal gate 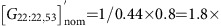 (Fig. 4c). The trajectories that compute logic ON reached an ideal high fluorescence state faster than the previous experiments shown in Fig. 4a and the trajectories that compute logic OFF remained at a lower fluorescence state that were roughly identical for all three input combinations, regardless of which upstream OR gate was ON. However, after identifying and adjusting the outlier gate, we still had a problem: the OFF trajectories were not at an ideal low fluorescence state. This led to the next tuning step that is necessary for unpurified seesaw circuits.

### Tuning circuit output

Comparing the behaviour of the AND gate when it was in isolation (Fig. 3d) and that when it was connected with two upstream OR gates (Fig. 4c), the ON/OFF separation was significantly decreased in the latter. These experimental results suggest that, compared with purified seesaw DNA circuits in which the ON/OFF separations were roughly identical from a single logic gate to four-layer logic circuits^12^, unpurified circuits are much noisier and the behaviour becomes less robust with more than one layer. We suspect this is caused by the stoichiometry errors in unpurified gate species. The double-stranded gate molecules were annealed with the same amount of top and bottom strands, because both strands have combinations of toehold and branch migration domains that can cause undesired interactions with other circuit components and thus neither should be in excess. However, due to variations in the pipetting volume and in the accuracy of concentrations, the equal stoichiometry cannot be guaranteed. Without purification, a small excess of one strand or another in the gate species cannot be removed. Therefore, the excess of strands would result in undesired release of output signals in logic gates, even without input signals, and introduce extra noise to downstream logic gates.

Fortunately, thanks to the thresholding function in every logic gate, we can tune the circuit output by increasing a threshold. A simple method for estimating how much threshold adjustment is needed is based on the ON/OFF separation of the circuit output. Using experimental data of a logic circuit with different inputs, we can choose a trajectory that should compute logic ON and OFF, respectively, and calculate the difference (*δ*) between the observed OFF value and an ideal OFF value, when the ON trajectory reaches an ideal ON value. Considering 0.7 and 0.3 as the lower bound, and 0.9 and 0.1 as the upper bound for an ideal ON/OFF separation, the range of *δ* can be determined as:


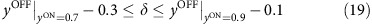


The nominal threshold in the logic gate that produces the circuit output can then be adjusted accordingly:


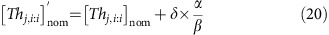


**Figure 5:**
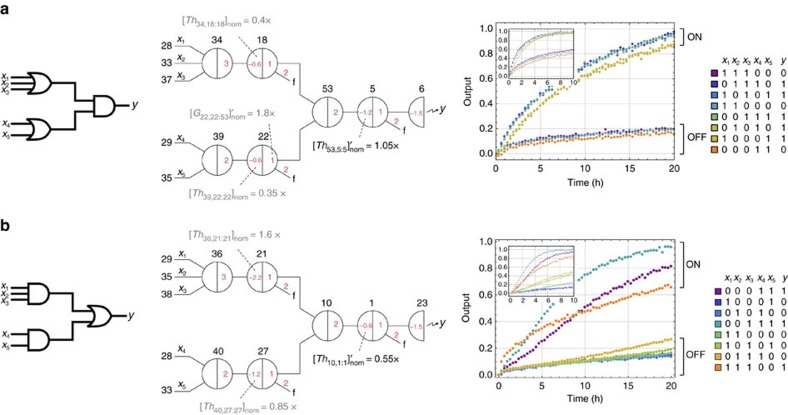
Tuning circuit output. Logic circuit diagram, seesaw circuit diagram and experimental data of a two-layer logic circuit with (**a**) two upstream OR gates connected to a downstream AND gate and (**b**) two upstream AND gates connected to a downstream OR gate. Nominal concentrations shown in grey and black indicate adjustments made in a previous step and in this step, respectively. Small insets of experimental data show the circuit behaviours before adjustments. 1 × =100 nM.

Using the data of the two-layer logic circuit shown in Fig. 4c, we chose the trajectory with input=01010 and 11100 as the reference ON and OFF trajectory, respectively, and calculated 0.08≤*δ*≤0.41. We then increased the threshold in the downstream AND gate to 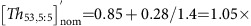 and repeated the experiment. The circuit behaviour was improved with a much better ON/OFF separation (Fig. 5a).

With the same method, we constructed another two-layer logic circuit that is composed of an OR gate and two upstream AND gates (Fig. 5b). In this case, using input=00011 and 01110 as the reference ON and OFF trajectories, we obtained a similar range of *δ* and decided to apply the same amount of increase to the threshold in the downstream OR gate.

It is noteworthy that a rule of thumb is to choose the slowest ON trajectory and the fastest OFF trajectory as the references for threshold adjustment, but different choices can be made if one has the knowledge of which data set is experimentally more reliable. Also note that increasing the threshold not only suppresses the OFF trajectories but also slows down the ON trajectories and thus this method of tuning the circuit output is only applicable if all ON trajectories are significantly faster than all OFF trajectories (which should be true if the thresholds and gates are properly calibrated).

Combining the two logic circuits shown in Fig. 5 and adding fan-out gates for input signals that are used in multiple logic gates, we successfully demonstrated the rule 124 sub-circuit consisting of 54 distinct DNA species (Supplementary Fig. 6).

We do not have evidence of how well unpurified circuits with multiple layers can be constructed, but we suspect that with the same amount of threshold increase (that is, *δ* × *α*/*β*) in all logic gates at layer two and above, undesired signals released from upstream gates can be effectively suppressed at every layer without accumulating over an increasing number of layers.

### Systematic procedure

Starting from the calibration of effective concentrations for threshold and gate species in general, to the identification and adjustment of any outliers, and then to the final tuning of circuit output, we established three sequential steps for building unpurified seesaw circuits. To make these steps easy to follow, we now further describe a systematic procedure, and evaluate the procedure by constructing a new logic circuit from scratch—the rule 110 sub-circuit.

**Figure 6:**
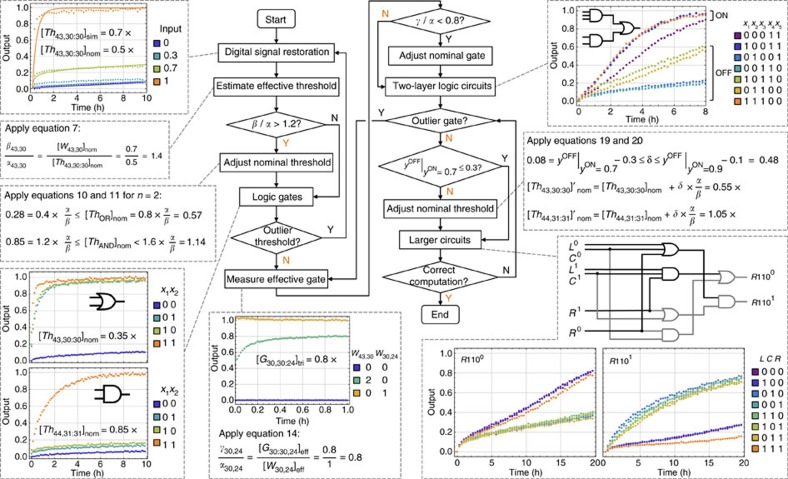
Flowchart for building seesaw DNA circuits using unpurified components. Insets show how the flowchart was used to construct the rule 110 sub-circuit. Y (yes) and N (no) highlighted in orange in the flowchart indicate the situations encountered and decisions made while building the rule 110 sub-circuit. 1 × =100 nM.

We summarized the procedure in a flowchart (Fig. 6). It starts with constructing the simplest functional component, digital signal restoration, and estimating the effective threshold compared to a signal. If the threshold to signal ratio *β*/*α*>1.2, adjust the nominal thresholds in all logic gates. Next, construct a single logic gate. If it fails to compute correctly, it indicates that the threshold species in this logic gate is an outlier, and thus one needs to go back to the first step and repeat the process to calibrate this particular threshold. Otherwise, move on to gate calibration experiments. If the gate to signal ratio *γ*/*α*<0.8, adjust all nominal gates.

Then construct a two-layer logic circuit, and identify if there exists an outlier gate. If so, repeat the process to calibrate this particular gate. At this point, the circuit still may not exhibit desired ON/OFF separation (for example, the OFF trajectories may be higher than 0.3 when the ON trajectories reach 0.7). However, if the ON trajectories are significantly faster than the OFF trajectories, increase the nominal threshold in the logic gate that directly produces the circuit output to tune the circuit behaviour. Continue to construct a larger circuit. If it fails to compute correctly, the most likely reason would be a new outlier gate. Identify the outlier based on cases where the ON/OFF separation is worst, and repeat the steps for calibrating the gate accordingly.

Following the flowchart, we completed the construction of the rule 110 sub-circuit in only 3 days (Fig. 6). If all components were PAGE purified, incrementally building the circuit would require at least one additional day for each new experiment, assuming no experimental errors. The turnaround time would be significantly increased.

**Figure 7:**
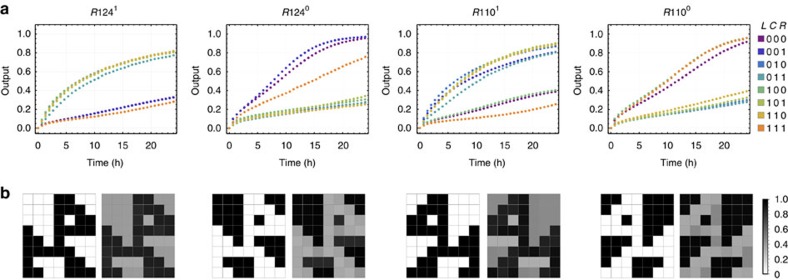
Implementing the rule 110–124 full circuit. (**a**) Fluorescence kinetics data of the two pairs of dual-rail outputs. 1 × =100 nM. All DNA sequences are listed in Supplementary Table 1. (**b**) Comparing the ideal logic circuit behaviour (left) with the DNA circuit behaviour (right). Each of the circuit outputs is illustrated by an array of 7 × 8 cells, representative of eight cellular automata generations on a torus with starting configuration (0,0,0,1,0,0,0). The arrays for the DNA circuit were plotted using the output values at 24 h from the data. The ideal logic circuit behaviour corresponds to an image of a black dog with a white background for *R*124^1^, an inverted image for *R*124^0^ and their mirror images for *R*110^1^ and *R*110^0^, respectively.

Combining the components from both rule 110 and rule 124 sub-circuits, using shared input-fanout gates and a three-input NAND gate (Fig. 2ab), the full rule 110–124 circuit consisting of 78 distinct DNA species was constructed in one test tube. The fluorescence kinetics experiments showed correct ON and OFF states of the two pairs of dual-rail outputs, for all eight possible inputs (Fig. 7a). To pictorially compare the ideal logic behaviour and the DNA circuit behaviour, we plotted each output into an array that represents eight cellular automata generations (Fig. 7b). The ideal logic circuit behaviour corresponds to four images of dogs. The DNA circuit behaviour yielded less contrast between the dogs and their backgrounds, but the patterns were still clearly recognizable.

### Modelling

Despite that the experiments were performed at a higher concentration (that is, 1 × =100 nM), the rule 110–124 circuit computed much slower than what the simulations predicted for 1 × =50 nM (Fig. 2c). We suspect that the difference was caused by the impurity of the molecules. To better predict the behaviour of seesaw circuits using unpurified components, we developed a model that takes synthesis errors into consideration.

We first define the probability of having *n* errors in a chemically synthesized DNA strand of *l* bases, given that *r* is the probability of synthesis error per base:


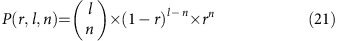


**Figure 8:**
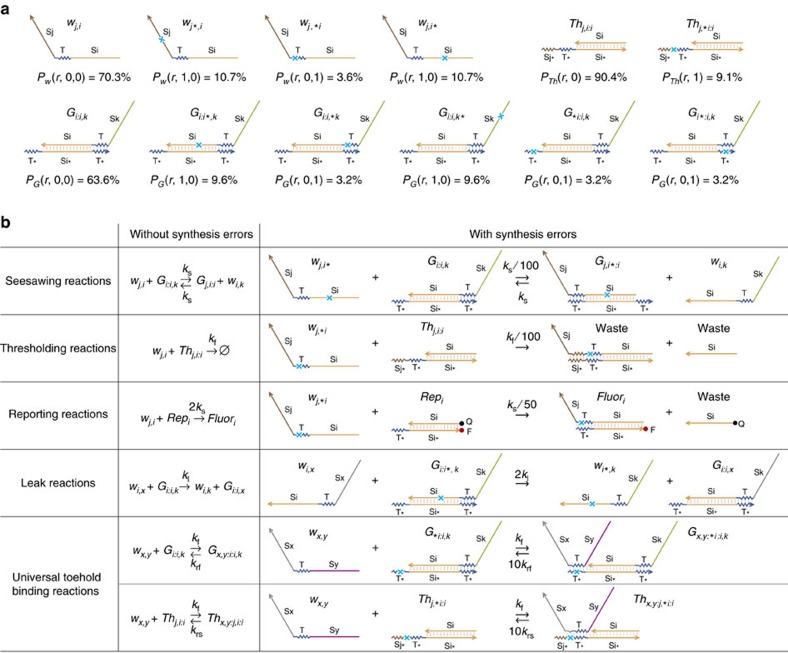
A model for unpurified seesaw circuits. (**a**) Populations of signal, gate and threshold molecules without and with synthesis errors in the marked locations. *r*=0.01. (**b**) Example reactions that involve DNA strands without and with synthesis errors. ∀*i*, *j*, *k*, *x* and *y*.

We then calculate the populations of signal, gate and threshold molecules with and without synthesis errors (Fig. 8a). To make the model simple enough, but accurate enough to describe reactions that involve molecules with synthesis errors at distinct locations, we treat the very small population of molecules with more than one synthesis error as non-reactive, and classify the remaining molecules containing a single synthesis error based on the domain where the error occurs. For example, a signal strand is composed of two branch migration domains flanking a toehold domain. Given that a branch migration domain has 15 bases and a toehold domain has 5 bases, the probability of a signal strand having *s* errors in a specific branch migration domain (and thus not in the other) and *t* errors in the toehold domain can be calculated as:


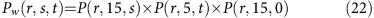


In a previous study on the robustness of a catalytic DNA strand displacement motif^21^, a single base mutation in an invading strand significantly slowed down (on the scale of 100 fold) a reversible strand displacement reaction that was designed with Δ*G*°≈0, both when the mutation was in the toehold and when it was in the branch migration domain. In contrast, an irreversible strand displacement reaction was only slowed down significantly (also on the scale of 100-fold) when the mutation was in the toehold domain, but the reaction rate remained roughly unchanged when the mutation was in the branch migration domain.

These observations lead us to the following interpretations: A synthesis error in the toehold domain can slow down strand displacement by increasing the disassociation rate of the toehold and thus decreasing the overall reaction rate. A synthesis error in the branch migration domain can also slow down strand displacement, but only when the energy change caused by the synthesis error is significant compared to the designed standard free energy of the reaction, and not when the reaction is already strongly favoured in one direction. Based on these interpretations, we estimated the rates of all five types of reactions in a seesaw network, involving all populations of defective molecules (Fig. 8b and Supplementary Note 1).

**Figure 9:**
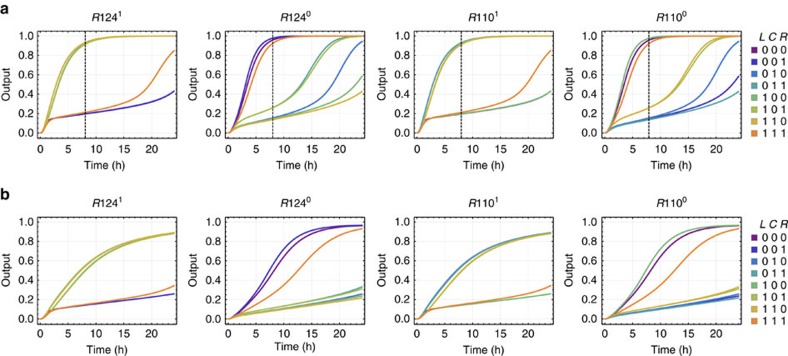
Simulations comparing the purified and unpurified models. (**a**) Simulations of the rule 110–124 circuit using the previously developed model for purified seesaw circuits, predicting that the circuit should yield desired outputs in roughly 8 h (shown as dotted lines) and the undesired reactions will take over in 24 h. (**b**) Simulations using the new model for unpurified seesaw circuits, predicting that the circuit should yield desired outputs in roughly 24 h. *k*_f_=2 × 10^6^ M^−1^ s^−1^, *k*_s_=5 × 10^4^ M^−1^ s^−1^, *k*_l_=10 M^−1^ s^−1^, *k*_rf_=26 s^−1^, *k*_rs_=1.3 s^−1^. 1 × =100 nM.

We first simulated the rule 110–124 circuit assuming that all molecules do not have synthesis errors, at the concentrations used in the experiments (Fig. 9a). Using exactly the same concentrations for all species, and the same rate parameters for reactions that are not affected by synthesis errors, we then simulated the circuit with each species divided into multiple populations including synthesis errors (Fig. 9b). The results of these two simulations were dramatically different: only the latter exhibited a remarkable degree of agreement with the data shown in Fig. 7a.

## Discussion

The biggest challenge that could prevent a molecular compiler from working in practice is that a new circuit may require new molecular components, which may not behave the same as the ones previously characterized. Thus, what made it possible to build a new complex circuit using the Seesaw Compiler? First, there are only three types of molecular components (signal, gate and threshold) for arbitrary feedforward logic circuits, which yield highly predictable circuit behaviour. Second, because of the simplicity of the molecules, there is minimal sequence design challenge. A three-letter code (A, T and C) for all signal strands is sufficient to eliminate undesired reactions. Finally, exact kinetics is not essential for qualitatively correct computation and thus small difference caused by DNA sequences should not affect the desired circuit behaviour.

On the other hand, the biggest challenge that could prevent us from using unpurified DNA strands is that the synthesis errors may lead to completely unpredictable molecular behaviours. Thus, what made it possible to build a complex circuit using unpurified strands? First, the Seesaw Compiler provides simulations as a debugging tool and makes it straightforward to identify problems caused by the synthesis errors. Second, again because there are only three types of species, it is relatively easy to understand the behaviours of defective molecules, as we expect similar synthesis quality across distinct species of the same type. More importantly, the signal restoration built in to every logic gate allows simple tuning to restore desired circuit behaviour, compensating for the impurity of molecules.

In general, there are several factors that we find important for the goals of producing a better molecular compiler, and implementing unpurified DNA circuits with more robust behaviours. Given that it is difficult to obtain fully predictable behaviour for newly designed molecular components, alternative architectures that enable arbitrary circuits to be created from a constant number of molecules will likely promote the development of compilers that work reliably in these contexts^31^. It is also necessary to eliminate leak reactions in DNA circuits^32^ and to improve the building blocks such that they are substantially less sensitive to synthesis errors and stoichiometry errors.

Nonetheless, with an experimental validation of the Seesaw Compiler and simplified experimental procedures using unpurified DNA strands described in this work, it is now possible to imagine a near future in which a molecular compiler can generate protocols from a high-level circuit function, and the protocols can then be executed by a liquid handling robot. Molecular engineers typing away on a computer to create biochemical circuits in a test tube is no longer just a distant dream.

## Methods

### DNA oligonucleotide synthesis

DNA oligonucleotides were purchased from Integrated DNA Technologies (IDT). The DNA strands in gate, threshold and fuel species were purchased unpurified (standard desalting). The reporter strands with fluorophores and quenchers were purchased purified (HPLC). All strands were purchased at 100 μM in TE buffer pH 8.0 and stored at 4 °C.

### Annealing protocol and buffer condition

Gate complexes were annealed together at 20 μM, with equal stoichiometry of top and bottom strands. Threshold and reporter complexes were annealed together at 20 μM with a 20% excess of top strands. All DNA complexes were annealed in 1 × TE buffer with 12.5 mM Mg^2+^, prepared from 100 × TE pH 8.0 (Fisher BioReagents) and 1 M MgCl_2_ (Invitrogen). Annealing was performed in a thermal cycler (Eppendorf), first heating up to 90 °C for 2 min and then slowly cooling down to 20 °C at the rate of 6 s per 0.1 °C. All annealed complexes were stored at 4 °C.

### Fluorescence spectroscopy

Fluorescence kinetics data in Figs 3, 4, 5, 6 and Supplementary Figs 3–6 were collected every 2 min in a monochromator-based plate reader (Synergy H1M, BioTek). Experiments were performed with 100 μl reaction mixture per well, in 96-well microplates (black with clear flat bottom, polystyrene NBS, Corning 3651) at 25 °C. Clear adhesive sealing tapes (Thermo Scientific Nunc 232701) were used to prevent evaporation. The excitation/emission wavelengths were set to 497/527 nm for ATTO 488 and 597/629 nm for ATTO 590.

Fluorescence kinetics data in Fig. 7 were collected every 4 min in a spectrofluorimeter (Fluorolog-3, Horiba). Experiments were performed with 500 μl reaction mixture per cuvette, in fluorescence cuvettes (Hellma 115 F-QS) at 25 °C. The excitation/emission wavelengths were set to 502/522 nm for ATTO 488, 602/624 nm for ATTO 590, 560/575 nm for ATTO 550 and 649/662 nm for ATTO 647. Both excitation and emission bandwidths were set to 2 nm and the integration time was 10 s for all experiments.

### Data analysis

A Mathematica Notebook file for data analysis and example data files are available to download at the Seesaw Compiler website: http://qianlab.caltech.edu/SeesawCompiler/DataAnalysis.php.

### Data availability

Key data supporting the findings of this study are available to download at the Seesaw Compiler website and all other data are available from the corresponding author upon reasonable request.

## Additional information

**How to cite this article:** Thubagere, A. J. *et al*. Compiler-aided systematic construction of large-scale DNA strand displacement circuits using unpurified components. *Nat. Commun.* **8,** 14373 doi: 10.1038/ncomms14373 (2017).

**Publisher's note**: Springer Nature remains neutral with regard to jurisdictional claims in published maps and institutional affiliations.

## Supplementary Material

Supplementary Information Supplementary Figures 1-6, Supplementary Table 1, Supplementary Note 1 and Supplementary References
