# Supplementary material for: Compiler-aided systematic construction of large-scale DNA strand displacement circuits using unpurified components
Source: Nat Commun. 2017 Feb 23;8:14373. doi: 10.1038/ncomms14373 (PMC5331218; doi:10.1038/ncomms14373)
Supplement: Supplementary Information — Supplementary Figures 1-6, Supplementary Table 1, Supplementary Note 1 and Supplementary References [file ncomms14373-s1.pdf]

Supplementary Figures

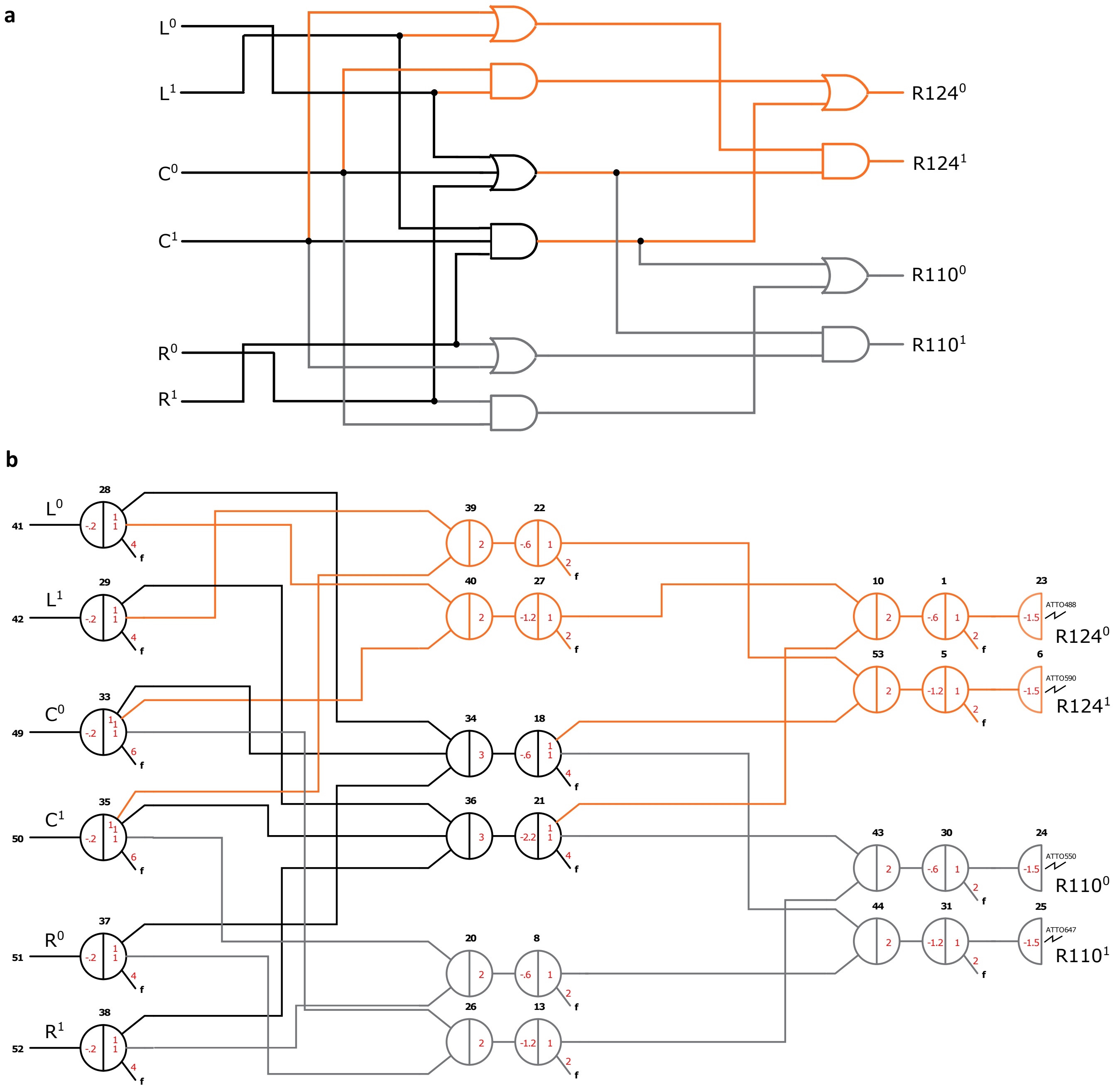

Supplementary Figure 1: **Diagrams of the rule 110-124 circuit. a,** Dual-rail circuit diagram. **b,** Seesaw circuit diagram.

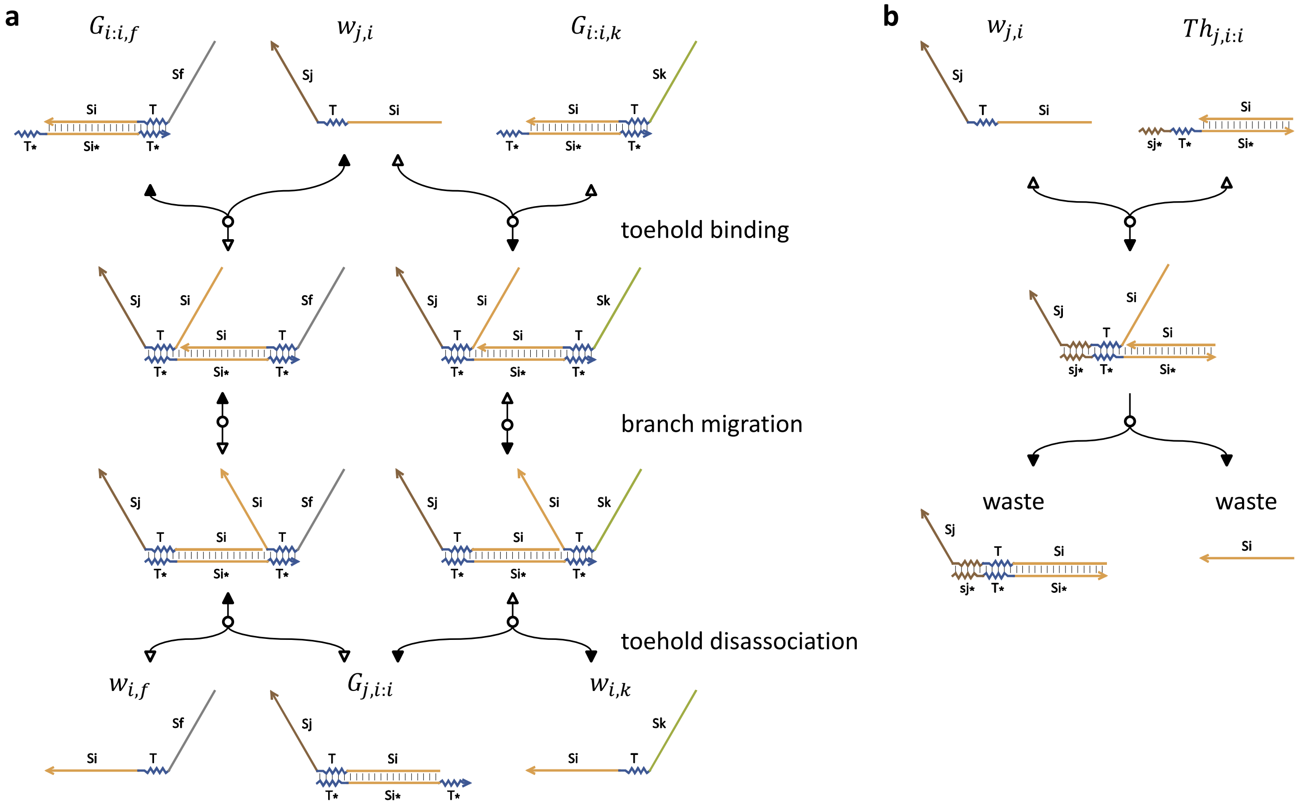

Supplementary Figure 2: **Basic DNA strand displacement reactions in a seesaw network** (adapted from ref.[1]). **a,** Catalysis. **b,** Thresholding. Solid arrows indicate flows of the forward reactions and outlined arrows indicate flows of the respective backward reactions. Catalysis is driven forward by a high concentration of the fuel species $w_{i,f}$ and downstream irreversible reactions (i.e. thresholding or reporting reactions) that consume the output species $w_{i,k}$. Matching colors and stars in domain names suggest complementary DNA sequences. For example, the blue domains T and $T*$ are complementary to each other, the orange domains Si and $Si*$ are complementary to each other, etc. $sj*$ is complementary to the first 5 nucleotides of the Sj domain. Thresholding is much faster than catalysis because the $sj*$ domain serves as an extended toehold, which significantly decreases the rate of toehold disassociation and thus speeds up the overall rate of stand displacement.[2,3]

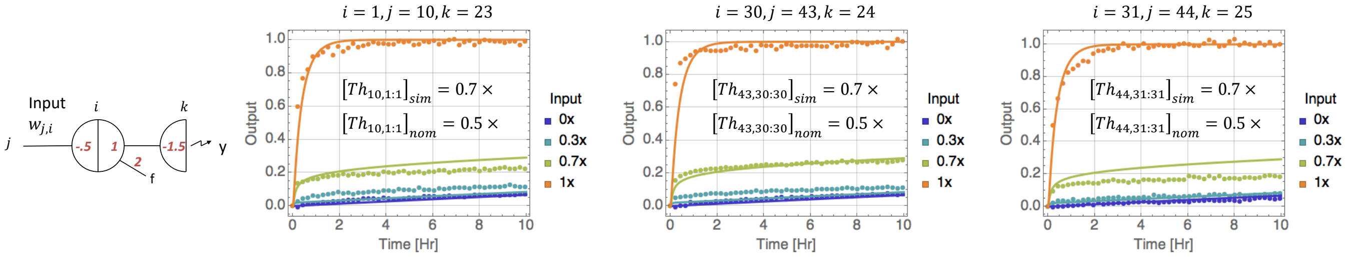

Supplementary Figure 3: **Estimating effective concentrations of distinct thresholds.** The small differences between simulations and data for $Th_{10,1:1}$ and $Th_{44,31:31}$ are considered non-significant. We show that $\beta/\alpha = 1.4$ works well enough for four distinct thresholds, including three shown here and one shown in Fig. 3c. $1\times = 100$ nM.

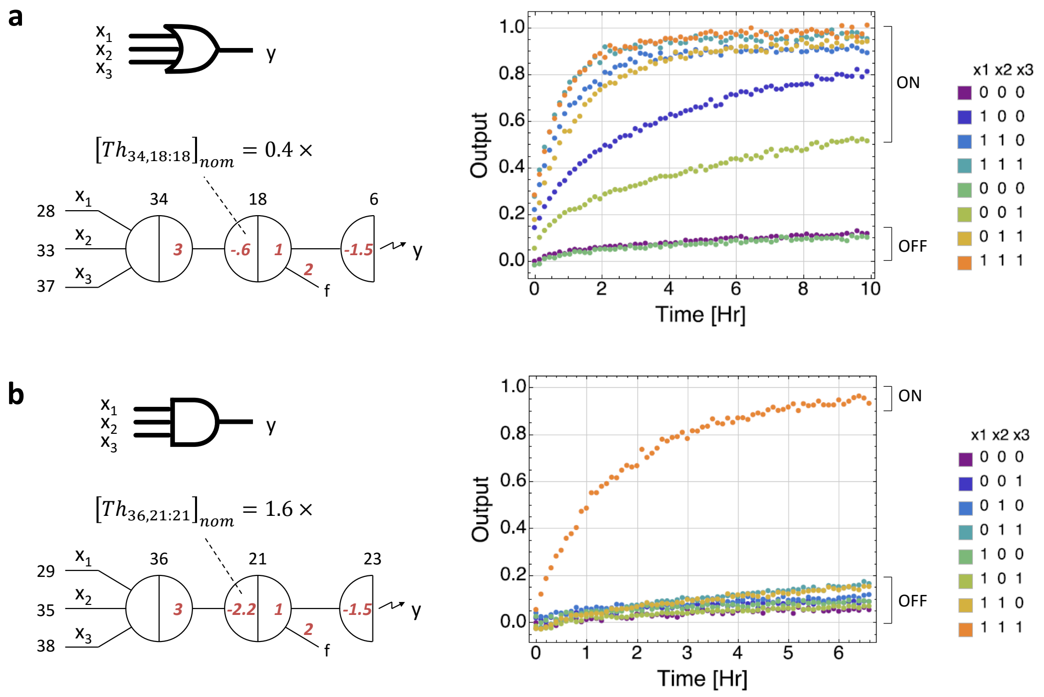

Supplementary Figure 4: **Three-input logic gates with adjusted nominal thresholds.** **a,** OR gate. Not all possible inputs were tested here ($x_1x_2x_3 = 000$ and 111 were repeated twice), but we believe that the circuit behavior for $x_1x_2x_3 = 010$ and 101 should be similar to that for 100/001 and 110/011, respectively. **b,** AND gate. $1\times = 100$ nM.

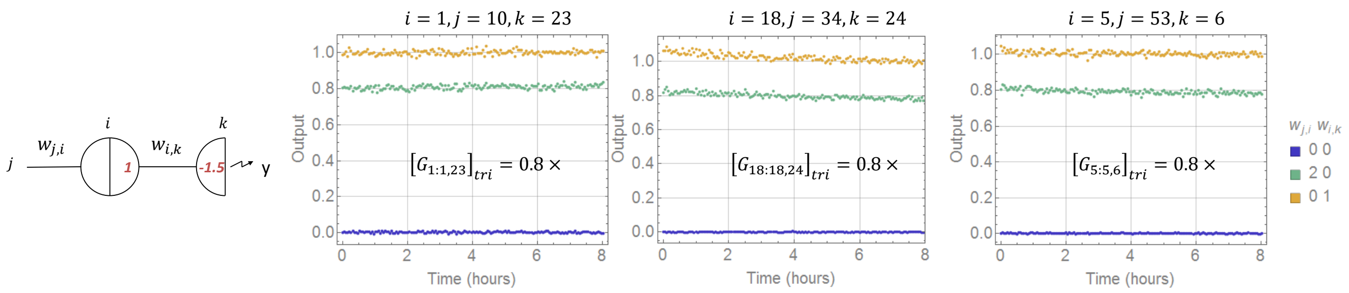

Supplementary Figure 5: **Estimating effective concentrations of distinct gates.** Data show steady state fluorescence level, as signal strands and gate molecules were mixed together and incubated before the measurements. We show that $\gamma/\alpha = 0.8$ works well for four distinct gates, including three shown here and one shown in Fig. 4b. $1\times = 100$ nM.

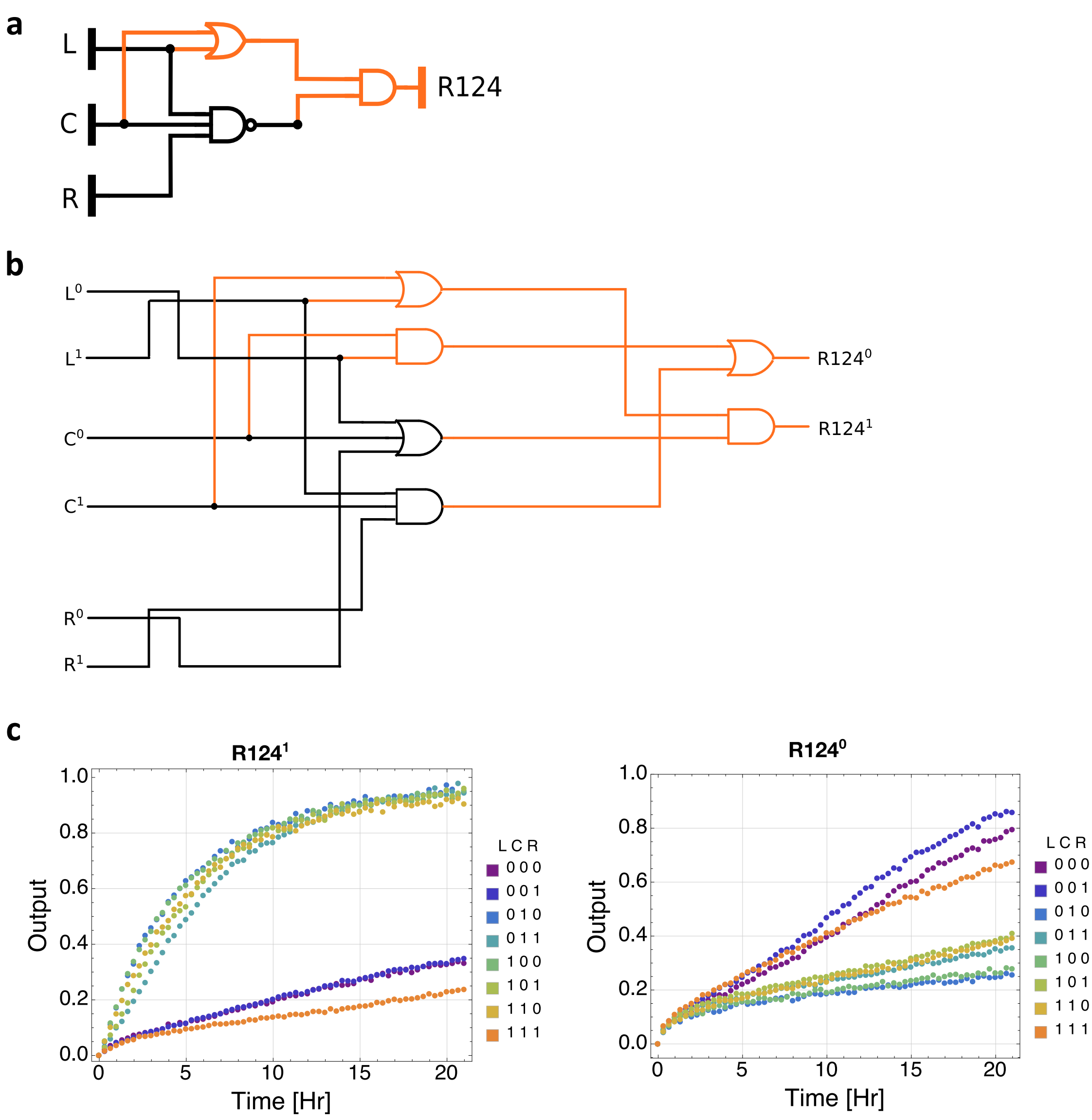

Supplementary Figure 6: **The rule 124 sub-circuit.** **a,** Logic circuit diagram. **b,** Dual-rail circuit diagram. **c,** Experimental data. $1\times = 100$ nM.

Supplementary Table 1

Supplementary Table 1: DNA sequences.

Name	Domain	Sequence
L^0: w41.28	S28 T S41	CATCTACAATTCACA TCT CAACAAACCATTACA
L^1: w42.29	S29 T S42	CACCAATACTCCTCA TCT CACTTTTCACTATCA
C^0: w49.33	S33 T S49	CAACTCAAACATACA TCT CATCCTTAACTCCCA
C^1: w50.35	S35 T S50	CACTCTCCATCACCA TCT CATTACCAACCACCA
R^0: w51.37	S37 T S51	CACCTCTTCCCTTCA TCT CACAAACTACATCCA
R^1: w52.38	S38 T S52	CATACCCTTTTCTCA TCT CACTTCACAACTACA
Th41.28:28-t	S28	CATCTACAATTCACA
Th41.28:28-b	s41* T* S28*	TTTGTTG AGA TGTGAATTGTAGATG
w28.34	S34 T S28	CACATAACAAAACCA TCT CATCTACAATTCACA
w28.40	S40 T S28	CAATACAAATCCACA TCT CATCTACAATTCACA
G28-b	T* S28* T*	TG AGA TGTGAATTGTAGATG AGA TG
w28.f	Sf T S28	CATTTTTTTTTTTCA TCT CATCTACAATTCACA
Th42.29:29-t	S29	CACCAATACTCCTCA
Th42.29:29-b	s42* T* S29*	AAAAGTG AGA TGAGGAGTATTGGTG
w29.36	S36 T S29	CAAACTAAACAACCA TCT CACCAATACTCCTCA
w29.39	S39 T S29	CACTATACACACCCA TCT CACCAATACTCCTCA
G29-b	T* S29* T*	TG AGA TGAGGAGTATTGGTG AGA TG
w29.f	Sf T S29	CATTTTTTTTTTTCA TCT CACCAATACTCCTCA
Th49.33:33-t	S33	CAACTCAAACATACA
Th49.33:33-b	s49* T* S33*	AAGGATG AGA TGTATGTTTGAGTTG
w33.34	S34 T S33	CACATAACAAAACCA TCT CAACTCAAACATACA
w33.40	S40 T S33	CAATACAAATCCACA TCT CAACTCAAACATACA
w33.26	S26 T S33	CATTCATTACCTCCA TCT CAACTCAAACATACA
G33-b	T* S33* T*	TG AGA TGTATGTTTGAGTTG AGA TG
w33.f	Sf T S33	CATTTTTTTTTTTCA TCT CAACTCAAACATACA
Th50.35:35-t	S35	CACTCTCCATCACCA
Th50.35:35-b	s50* T* S35*	GGTAATG AGA TGGTGATGGAGAGTG
w35.36	S36 T S35	CAAACTAAACAACCA TCT CACTCTCCATCACCA
w35.39	S39 T S35	CACTATACACACCCA TCT CACTCTCCATCACCA
w35.20	S20 T S35	CAATCTAACACTCCA TCT CACTCTCCATCACCA
G35-b	T* S35* T*	TG AGA TGGTGATGGAGAGTG AGA TG
w35.f	Sf T S35	CATTTTTTTTTTTCA TCT CACTCTCCATCACCA
Th51.37:37-t	S37	CACCTCTTCCCTTCA
Th51.37:37-b	s51* T* S37*	GTTTGTG AGA TGAAGGGAAGAGGTG
w37.34	S34 T S37	CACATAACAAAACCA TCT CACCTCTTCCCTTCA
w37.26	S26 T S37	CATTCATTACCTCCA TCT CACCTCTTCCCTTCA
G37-b	T* S37* T*	TG AGA TGAAGGGAAGAGGTG AGA TG
w37.f	Sf T S37	CATTTTTTTTTTTCA TCT CACCTCTTCCCTTCA
Th52.38:38-t	S38	CATACCCTTTTCTCA
Th52.38:38-b	s52* T* S38*	TGAAGTG AGA TGAGAAAAGGGTATG
w38.36	S36 T S38	CAAACTAAACAACCA TCT CATACCCTTTTCTCA
w38.20	S20 T S38	CAATCTAACACTCCA TCT CATACCCTTTTCTCA
G38-b	T* S38* T*	TG AGA TGAGAAAAGGGTATG AGA TG
w38.f	Sf T S38	CATTTTTTTTTTTCA TCT CATACCCTTTTCTCA
w34.18	S18 T S34	CATCTTCTAACATCA TCT CACATAACAAAACCA
G34-b	T* S34* T*	TG AGA TGGTTTTGTTATGTG AGA TG
Th34.18:18-t	S18	CATCTTCTAACATCA
Th34.18:18-b	s34* T* S18*	TTATGTG AGA TGATGTTAGAAGATG
w18.53	S53 T S18	CATATCTAATCTCCA TCT CATCTTCTAACATCA
w18.44	S44 T S18	CAAAACTCTCTCTCA TCT CATCTTCTAACATCA

Name	Domain	Sequence
G18-b	T* S18* T*	TG AGA TGATGTTAGAAGATG AGA TG
w18.f	Sf T S18	CATTTTTTTTTTTCA TCT CATCTTCTAACATCA
w36.21	S21 T S36	CAACCATACTAAACA TCT CAAACTAAACAACCA
G36-b	T* S36* T*	TG AGA TGGTTGTTTAGTTTG AGA TG
Th36.21:21-t	S21	CAACCATACTAAACA
Th36.21:21-b	s36* T* S21*	TAGTTTG AGA TGTTTAGTATGGTTG
w21.10	S10 T S21	CATACAACATCTACA TCT CAACCATACTAAACA
w21.43	S43 T S21	CATCATACCTACTCA TCT CAACCATACTAAACA
G21-b	T* S21* T*	TG AGA TGTTTAGTATGGTTG AGA TG
w21.f	Sf T S21	CATTTTTTTTTTTCA TCT CAACCATACTAAACA
w26.13	S13 T S26	CACAACTCATTACCA TCT CATTCATTACCTCCA
G26-b	T* S26* T*	TG AGA TGGAGGTAATGAATG AGA TG
Th26.13:13-t	S13	CACAACTCATTACCA
Th26.13:13-b	s26* T* S13*	ATGAATG AGA TGGTAATGAGTTGTG
w13.43	S43 T S13	CATCATACCTACTCA TCT CACAACTCATTACCA
G13-b	T* S13* T*	TG AGA TGGTAATGAGTTGTG AGA TG
w13.f	Sf T S13	CATTTTTTTTTTTCA TCT CACAACTCATTACCA
w20.8	S8 T S20	CACTAACATACAACA TCT CAATCTAACACTCCA
G20-b	T* S20* T*	TG AGA TGGAGTGTTAGATTG AGA TG
Th20.8:8-t	S8	CACTAACATACAACA
Th20.8:8-b	s20* T* S8*	TAGATTG AGA TGTTGTATGTTAGTG
w8.44	S44 T S8	CAAAACTCTCTCTCA TCT CACTAACATACAACA
G8-b	T* S8* T*	TG AGA TGTTGTATGTTAGTG AGA TG
w8.f	Sf T S8	CATTTTTTTTTTTCA TCT CACTAACATACAACA
w43.30	S30 T S43	CACCATTACAATCCA TCT CATCATACCTACTCA
G43-b	T* S43* T*	TG AGA TGAGTAGGTATGATG AGA TG
Th43.30:30-t	S30	CACCATTACAATCCA
Th43.30:30-b	s43* T* S30*	TATGATG AGA TGGATTGTAATGGTG
w30.24	S24 T S30	CACTCATCCTTTACA TCT CACCATTACAATCCA
G30-b	T* S30* T*	TG AGA TGGATTGTAATGGTG AGA TG
w30.f	Sf T S30	CATTTTTTTTTTTCA TCT CACCATTACAATCCA
w44.31	S31 T S44	CAATCCACACTTCCA TCT CAAAACTCTCTCTCA
G44-b	T* S44* T*	TG AGA TGAGAGAGAGTTTTG AGA TG
Th44.31:31-t	S31	CAATCCACACTTCCA
Th44.31:31-b	s44* T* S31*	AGTTTTG AGA TGGAAGTGTGGATTG
w31.25	S25 T S31	CAATTCACTCAATCA TCT CAATCCACACTTCCA
G31-b	T* S31* T*	TG AGA TGGAAGTGTGGATTG AGA TG
w31.f	Sf T S31	CATTTTTTTTTTTCA TCT CAATCCACACTTCCA
w40.27	S27 T S40	CAAACACTCTATTCA TCT CAATACAAATCCACA
G40-b	T* S40* T*	TG AGA TGTGGATTTGTATTG AGA TG
Th40.27:27-t	S27	CAAACACTCTATTCA
Th40.27:27-b	s40* T* S27*	TGTATTG AGA TGAATAGAGTGTTTG
w27.10	S10 T S27	CATACAACATCTACA TCT CAAACACTCTATTCA
G27-b	T* S27* T*	TG AGA TGAATAGAGTGTTTG AGA TG
w27.f	Sf T S27	CATTTTTTTTTTTCA TCT CAAACACTCTATTCA
w39.22	S22 T S39	CATTCCTACATTTCA TCT CACTATACACACCCA
G39-b	T* S39* T*	TG AGA TGGGTGTGTATAGTG AGA TG
Th39.22:22-t	S22	CATTCCTACATTTCA
Th39.22:22-b	s39* T* S22*	TATAGTG AGA TGAAATGTAGGAATG
w22.53	S53 T S22	CATATCTAATCTCCA TCT CATTCCTACATTTCA
G22-b	T* S22* T*	TG AGA TGAAATGTAGGAATG AGA TG
w22.f	Sf T S22	CATTTTTTTTTTTCA TCT CATTCCTACATTTCA
w10.1	S1 T S10	CATCCATTCCACTCA TCT CATACAACATCTACA

Name	Domain	Sequence
G10-b	T* S10* T*	TG AGA TGTAGATGTTGTATG AGA TG
Th10.1:1-t	S1	CATCCATTCCACTCA
Th10.1:1-b	s10* T* S1*	TTGTATG AGA TGAGTGGAATGGATG
w1.23	S23 T S1	CAAATCTTCATCCCA TCT CATCCATTCCACTCA
G1-b	T* S1* T*	TG AGA TGAGTGGAATGGATG AGA TG
w1.f	Sf T S1	CATTTTTTTTTTTCA TCT CATCCATTCCACTCA
w53.5	S5 T S53	CACCACCAAACTTCA TCT CATATCTAATCTCCA
G53-b	T* S53* T*	TG AGA TGGAGATTAGATATG AGA TG
Th53.5:5-t	S5	CACCACCAAACTTCA
Th53.5:5-b	s53* T* S5*	AGATATG AGA TGAAGTTTGGTGGTG
w5.6	S6 T S5	CATAACACAATCACA TCT CACCACCAAACTTCA
G5-b	T* S5* T*	TG AGA TGAAGTTTGGTGGTG AGA TG
w5.f	Sf T S5	CATTTTTTTTTTTCA TCT CACCACCAAACTTCA
Rep6-t	RQ S6	/5IAbRQ/ CATAACACAATCACA
Rep6-b	T* S6* ATTO590	TG AGA TGTGATTGTGTTATG /3ATTO590N/
Rep23-t	FQ S23	/5IABkFQ/ CAAATCTTCATCCCA
Rep23-b	T* S23* ATTO488	TG AGA TGGGATGAAGATTTG /3ATTO488N/
Rep24-t	RQ S24	/5IAbRQ/ CACTCATCCTTTACA
Rep24-b	T* S24* ATTO550	TG AGA TGTAAAGGATGAGTG /3ATTO550N/
Rep25-t	RQ S25	/5IAbRQ/ CAATTCACTCAATCA
Rep25-b	T* S25* ATTO647	TG AGA TGATTGAGTGAATTG /3ATTO647NN/

Supplementary Note 1

1 Modeling

1.1 Molecules with synthesis errors

We first define the probability of having n errors in a chemically synthesized DNA strand of l bases, given that r is the probability of synthesis error per base:

$$P(r,l,n) = \binom{l}{n} \times (1-r)^{l-n} \times r^n \tag{1}$$

We then calculate the populations of signal, gate and threshold molecules with and without synthesis errors (Fig. 8a). To make the model simple enough, but accurate enough to describe reactions that involve molecules with synthesis errors at distinct locations, we treat the very small population of molecules with more than one synthesis error as non-reactive, and classify the remaining molecules containing a single synthesis error based on the domain where the error occurs. For example, a signal strand is composed of two branch migration domains flanking a toehold domain (Fig. 8a, top left). Given that a branch migration domain has 15 bases and a toehold domain has 5 bases, the probability of a signal strand having s errors in a specific branch migration domain (and thus not in the other) and t errors in the toehold domain can be calculated as:

$$P_w(r,s,t) = P(r,15,s) \times P(r,5,t) \times P(r,15,0) \tag{2}$$

It is known that the failure rate for each nucleotide coupling event during DNA synthesis is 1% or less[4] (`https://www.idtdna.com/pages/docs/technical-reports/chemical-synthesis-of-oligonucleotides.pdf`), and we choose to use $r = 0.01$ in all following calculations. Specifically, a signal species composed of domains Sj, T and Si can be classified into five populations: $P_w(r,0,0) = 70.3\%$ with no synthesis errors (named $w_{j,i}$), $P_w(r,1,0) = 10.7\%$ with an error in the Sj domain (named $w_{j*,i}$), $P_w(r,0,1) = 3.6\%$ with an error in the T domain (named $w_{j,*i}$), $P_w(r,1,0) = 10.7\%$ with an error in the Si domain (named $w_{j,i*}$), and $1 - P_w(r,0,0) - 2 \times P_w(r,1,0) - P_w(r,0,1) = 4.8\%$ with two or more errors (considered as inert and not participating in any reactions). The location of a star in the name corresponds to the location of a synthesis error. Because the same toehold domain (that we call the universal toehold) is used in all signal species and thus not specified in the name, an error in the toehold domain is indicated by a star following the comma that separates j and i.

A gate molecule consists of a signal strand bound to a gate bottom strand that has two toehold domains flanking a branch migration domain (Fig. 8a, bottom). The gate bottom strand is never free, and only participates in reactions of two signal strands competing for the same bottom strand. Any error in the branch migration domain of the bottom strand should not significantly affect the reaction rate, because it does not bias the competition in either direction, and after the initiation of strand displacement, random walk steps of adjacent base pair opening and closing should remain sufficiently fast.[3] Thus we only consider errors in the remaining two branch migration domains and three toehold domains. The probability of a gate molecule having s errors in a specific branch migration domain (and thus not the other) and t errors in a specific toehold domain (and thus not the other two) can be calculated as:

$$P_G(r,s,t) = P(r,15,s) \times P(r,5,t) \times P(r,25,0) \tag{3}$$

A threshold molecule consists of an extended toehold domain of 10 bases and two complementary branch migration domains (Fig. 8a, top right). The branch migration domains only participate in irreversible strand displacement reactions of a threshold molecule consuming a signal strand. An error in these two domains should not significantly affect the reaction rate, because it either occur in the top strand and bias the reaction more forward, which has little effect since the reaction is already strongly favored in the forward direction, or occur in the bottom strand and doesn't introduce additional bias to the random walk steps. Thus we only consider errors in the extended toehold domain. The probability of a threshold molecule having t errors can be calculated as:

$$P_{Th}(r,t) = P(r,10,t) \tag{4}$$

Using equations 3 and 4, a gate species can be classified into seven populations, including $1 - P_G(r,0,0) - 2 \times P_G(r,1,0) - 3 \times P_G(r,0,1) = 7.5\%$ inert molecules. A threshold species can be classified into three populations, including $1 - P_{Th}(r,0) - P_{Th}(r,1) = 0.4\%$ inert molecules.

1.2 Reactions that involve molecules with synthesis errors

Seesaw circuits can be modeled with five types of reactions[1] (Fig. 8b): seesawing reactions that reversibly exchange two signals between inactive (i.e. bound to a gate) and active (i.e. free-floating) states, thresholding reactions that irreversibly consume a signal, reporting reactions that generate fluorescence readout, leak reactions that slowly release a signal from a gate molecule, and universal toehold binding reactions that temporarily occur between any single strand and any gate or threshold molecules. Compared to the reactions that only involve molecules without synthesis errors, there is a much longer list of reactions that involve molecules with synthesis errors, because each distinct species is now divided into multiple populations. To concisely describe these reactions, we define that reactions in the following format

$$\{R_{11}, R_{12}, \cdots, R_{1n}\} + \{R_{21}, R_{22}, \cdots, R_{2m}\} \xrightarrow{k} \{P_{11}, P_{12}, \cdots, P_{1n}\} + \{P_{21}, P_{22}, \cdots, P_{2m}\} \tag{5}$$

can be interpreted as the set of reactions:

$$R_{1i} + R_{2j} \xrightarrow{k} P_{1i} + P_{2j}, \forall 1 \leq i \leq n, 1 \leq j \leq m \tag{6}$$

This means for reactions with two reactants and two products, we always group them together in a way that the first product can be determined just based on the first reactant, and the second product can be determined just based on the second reactant. Note that a reversible reaction can be seen as two irreversible reactions that each follow the same definition.

Based on the previous results,[5] we estimated the rates of seesawing and thresholding reactions that involve all populations of signal, gate and threshold molecules shown in Fig. 8a.

(1) Seesawing reactions:

If there is no error in the domains participating in a seesawing reaction, regardless of any errors in the other domains (e.g. Sj and Sk domains for $w_{j,i}$ interacts with $G_{i:i,k}$), the rate remains the same as in the previous model for purified seesaw circuits.

$$\{w_{j,i}, w_{j*,i}\} + \{G_{i:i,k}, G_{i:i,k*}\} \underset{k_s}{\overset{k_s}{\rightleftharpoons}} \{G_{j,i:i}, G_{j*,i:i}\} + \{w_{i,k}, w_{i,k*}\} \tag{7}$$

If there is an error in the participating toehold or branch migration domain of the invading signal strand, or in the initiating toehold domain of the gate molecule (i.e. the toehold that binds to the invading signal strand), the forward rate is 100 times slower and the backward rate remains the same.

$$\begin{aligned} \{w_{j,*i}, w_{j,i*}\} + \{G_{i:i,k}, G_{i:i,k*}\} &\underset{k_s}{\overset{k_s/100}{\rightleftharpoons}} \{G_{j,*i:i}, G_{j,i*:i}\} + \{w_{i,k}, w_{i,k*}\} \\ \{w_{j,i}, w_{j*,i}\} + \{G_{*i:i,k}, G_{*i:i,k*}\} &\underset{k_s}{\overset{k_s/100}{\rightleftharpoons}} \{G_{j,i:*i}, G_{j*,i:*i}\} + \{w_{i,k}, w_{i,k*}\} \end{aligned} \tag{8}$$

Symmetrically, if there is an error in the participating toehold or branch migration domain of the bound signal in the gate molecule, or in the disassociation toehold domain (i.e. the toehold that is originally covered), the backward rate is 100 times slower and the forward rate remains the same.

$$\begin{aligned} \{w_{j,i}, w_{j*,i}\} + \{G_{i:i*,k}, G_{i:i,*k}\} &\underset{k_s/100}{\overset{k_s}{\rightleftharpoons}} \{G_{j,i:i}, G_{j*,i:i}\} + \{w_{i*,k}, w_{i,*k}\} \\ \{w_{j,i}, w_{j*,i}\} + \{G_{i*:i,k}, G_{i*:i,k*}\} &\underset{k_s/100}{\overset{k_s}{\rightleftharpoons}} \{G_{j,i:i*}, G_{j*,i:i*}\} + \{w_{i,k}, w_{i,k*}\} \end{aligned} \tag{9}$$

Note that gate molecules with two synthesis errors (e.g. $G_{*i:i,k*}$ and $G_{i*:i,k*}$) are not in the initial populations but can be produced by a seesawing reaction between signal and gate molecules that each have just one synthesis error. Reactions are omitted if there exist more than one synthesis error that can significantly affect the rate, because they are either too slow or do not have enough reactants to take place. For example,

$$w_{j,i*} + G_{*i:i,k} \underset{k_s}{\overset{\ll k_s/100}{\rightleftharpoons}} G_{j,i*:*i} + w_{i,k}$$

and

$$w_{j,i*} + G_{i:i*,k} \underset{k_s/100}{\overset{k_s/100}{\rightleftharpoons}} G_{j,i*:i} + w_{i*,k}$$

(2) Thresholding reactions:

Unlike a seesawing reaction, if there is no error in the toehold domains participating in a thresholding reaction, regardless of any errors in the branch migration domains, the rate remains the same as in the previous model for purified seesaw circuits.

$$\{w_{j,i}, w_{j*,i}, w_{j,i*}\} + Th_{j,i:i} \xrightarrow{k_f} \emptyset \tag{10}$$

Otherwise the rate is 100 times slower.

$$\begin{aligned} w_{j,*i} + Th_{j,i:i} &\xrightarrow{k_f/100} \emptyset \\ \{w_{j,i}, w_{j*,i}, w_{j,i*}\} + Th_{j,*i:i} &\xrightarrow{k_f/100} \emptyset \end{aligned} \tag{11}$$

Reactions are again omitted if there exist more than one synthesis error that can significantly affect the rate. For example,

$$w_{j,*i} + Th_{j,*i:i} \xrightarrow{\ll k_f/100} \emptyset$$

An error in the extended toehold but not in the universal toehold domain of a signal strand is not considered to affect the rate of thresholding, because an error more distant from the branch migration domain should affect the rate less, and considering it would complicate the classification of signal molecules.

(3) Reporting reactions:

Reporting reactions are also irreversible, and thus are modeled similarly as the thresholding reactions, based on if there is an error in the toehold domains.

$$\begin{aligned} \{w_{j,i}, w_{j*,i}, w_{j,i*}\} + Rep_i &\xrightarrow{2k_s} Fluor_i \\ w_{j,*i} + Rep_i &\xrightarrow{k_s/50} Fluor_i \end{aligned} \tag{12}$$

(4) Leak reactions:

Leak reactions are essentially 0-toehold strand displacement reactions. If there is no error in the participating domains of the two competing signal strands, the rate remains the same as in the previous model for purified seesaw circuits. An error in the gate bottom strand should not affect the rate significantly, regardless of if it is in the toehold domain, because the toehold is covered and thus treated the same as the branch migration domain.

$$\begin{aligned} \{w_{i,x}, w_{i,x*}\} + \{G_{i:i,k}, G_{i:i,k*}\} &\xrightarrow{k_l} \{G_{i:i,x}, G_{i:i,x*}\} + \{w_{i,k}, w_{i,k*}\} \\ \{w_{i,x}, w_{i,x*}\} + \{G_{*i:i,k}, G_{*i:i,k*}\} &\xrightarrow{k_l} \{G_{*i:i,x}, G_{*i:i,x*}\} + \{w_{i,k}, w_{i,k*}\} \\ \{w_{i,x}, w_{i,x*}\} + \{G_{i*:i,k}, G_{i*:i,k*}\} &\xrightarrow{k_l} \{G_{i*:i,x}, G_{i*:i,x*}\} + \{w_{i,k}, w_{i,k*}\} \end{aligned} \tag{13}$$

Leak reactions should be faster, if there is an error in the participating toehold or branch migration domain of the bound signal strand in the gate molecule, because the forward reaction will be favored. The reaction would be roughly 10 times faster if the error occurs at either end of the double-stranded domain and opens up a 1-nucleotide toehold for the invading signal strand. However, the error should occur in the middle of the double-stranded domain, with a much higher probability, and thus serve as a much less effective 1-nucleotide toehold. Therefore, we estimate the rate to be only twice as fast.

$$\begin{aligned} \{w_{i,x}, w_{i,x*}\} + \{G_{i:i*,k}, G_{i:i,*k}\} &\xrightarrow{2k_l} \{G_{i:i,x}, G_{i:i,x*}\} + \{w_{i*,k}, w_{i,*k}\} \\ \{w_{i,x}, w_{i,x*}\} + \{G_{*i:i*,k}, G_{*i:i,*k}\} &\xrightarrow{2k_l} \{G_{*i:i,x}, G_{*i:i,x*}\} + \{w_{i*,k}, w_{i,*k}\} \\ \{w_{i,x}, w_{i,x*}\} + \{G_{i*:i*,k}, G_{i*:i,*k}\} &\xrightarrow{2k_l} \{G_{i*:i,x}, G_{i*:i,x*}\} + \{w_{i*,k}, w_{i,*k}\} \end{aligned} \tag{14}$$

Since leak reactions are already very slow, reactions are omitted if there is an error that slows down the rate even further. For example,

$$w_{i*,x} + G_{i:i,k} \xrightarrow{\ll k_l} G_{i:i*,x} + w_{i,k}$$

(5) Universal toehold binding reactions:

Finally, the forward rate remains the same for all universal toehold binding reactions, since it is just the rate of hybridization. The backward rate remains the same if there is no error in the toehold domains, and is 10 times faster if there is an error, simply because the rate of toehold disassociation can be estimated as 10^{6-l} /s, where l is the number of bases in the toehold.[2,3]

$$\begin{gathered}
W + \{G_{i:i,k}, G_{i*:i,k}, G_{i:i*,k}, G_{i:i,*k}, G_{i:i,k*}, G_{i*:i,k*}\} \underset{k_{rf}}{\overset{k_f}{\rightleftharpoons}} \{G_{W:i:i,k}, G_{W:i*:i,k}, G_{W:i:i*,k}, G_{W:i:i,*k}, G_{W:i:i,k*}, G_{W:i*:i,k*}\} \\
W + \{G_{*i:i,k}, G_{*i:i,k*}\} \underset{10k_{rf}}{\overset{k_f}{\rightleftharpoons}} \{G_{W:*i:i,k}, G_{W:*i:i,k*}\} \\
\{G_{j,i:i}, G_{j*,i:i}, G_{j,*i:i}, G_{j,i*:i}, G_{j,i:*i}, G_{j*,i:*i}\} + W \underset{k_{rf}}{\overset{k_f}{\rightleftharpoons}} \{G_{j,i:i:W}, G_{j*,i:i:W}, G_{j,*i:i:W}, G_{j,i*:i:W}, G_{j,i:*i:W}, G_{j*,i:*i:W}\} \\
\{G_{j,i:i*}, G_{j*,i:i*}\} + W \underset{10k_{rf}}{\overset{k_f}{\rightleftharpoons}} \{G_{j,i:i*:W}, G_{j*,i:i*:W}\} \\
W + Th_{j,i:i} \underset{k_{rs}}{\overset{k_f}{\rightleftharpoons}} Th_{W:j,i:i} \\
W + Th_{j,*i:i} \underset{10k_{rs}}{\overset{k_f}{\rightleftharpoons}} Th_{W:j,*i:i} \\
\{w_{j,i}, w_{j*,i}, w_{j,i*}\} + G \underset{k_{rf}}{\overset{k_f}{\rightleftharpoons}} \{G_{j,i:G}, G_{j*,i:G}, G_{j,i*:G}\} \\
w_{j,*i} + G \underset{10k_{rf}}{\overset{k_f}{\rightleftharpoons}} G_{j,*i:G} \\
\{w_{j,i}, w_{j*,i}, w_{j,i*}\} + TH \underset{k_{rs}}{\overset{k_f}{\rightleftharpoons}} \{TH_{j,i:TH}, TH_{j*,i:TH}, TH_{j,i*:TH}\} \\
w_{j,*i} + TH \underset{10k_{rs}}{\overset{k_f}{\rightleftharpoons}} TH_{j,*i:TH}
\end{gathered} \tag{15}$$

$$\begin{aligned}
[W]|_{t=0} &= \sum [w_{j,i}]|_{t=0}, \forall i, j \\
[G]|_{t=0} &= \sum [G_{j,i:i}]|_{t=0} + [G_{i:i,k}]|_{t=0} + [Rep_i]|_{t=0}, \forall i, j, k \\
[TH]|_{t=0} &= \sum [Th_{j,i:i}]|_{t=0}, \forall i, j
\end{aligned} \tag{16}$$

1.3 Approximation in domain lengths

When calculating the populations of molecules with synthesis errors in distinct domains, we assume that a branch migration domain has 15 bases, a toehold domain has 5 bases, and a signal strand is composed of two branch migration domains flanking a toehold domain. This is a simplification of the actual components of a signal strand. To reduce undesired leak reactions between two gate species, a signal strand is designed to include clamp domains of 2 bases (more details see supplementary notes S8 of ref.[1]). These 2 bases are either part of a branch migration domain, or part of a toehold domain, depending on which side of a gate the signal strand is bound to or interact with. Because of the double identities of a clamp domain, a signal strand actually has 33 bases. Considering the clamp domains would significantly complicate the classification of molecules and reactions, and would only result in a very small difference compared to the calculations that we made in the model. Thus, we chose to not consider the clamp domains and used the approximation that a signal strand has 35 bases.

1.4 Concentrations of threshold species

Our model including molecules with synthesis errors explained the significant slow down of the unpurified seesaw circuits compared to purified ones, but it cannot explain why the threshold molecules had significantly higher effective concentrations compared to the signal strands. In fact, we applied threshold to signal ratio $\beta/\alpha = 1.4$, as calculated

in equation 7, to all threshold concentrations in the simulations. Having higher threshold concentrations in the model was actually not new to seesaw circuits. An $\beta/\alpha = 1.1$ was applied to all threshold concentrations in the previously-developed model for purified seesaw circuits.[1] Unlike how we specifically measured β/α in this work, the $1.1\times$ nominal threshold was simply tuned to obtain a better agreement between simulations and experiments for large circuits. We suspect that the difference in concentrations of threshold and signal species (both free-floating and bound to a gate), is caused by certain aspects of the DNA oligonucleotide synthesis procedures that we do not yet understand. This difference may be improved (e.g. from $1.4\times$ to $1.1\times$), but cannot be completely removed, by in-house gel purification. Thus, it is important that the value of β/α is determined by users of the Seesaw Compiler, following the procedures that we discussed in threshold calibration.

Supplementary References

[1] Qian, L. & Winfree, E. Scaling up digital circuit computation with DNA strand displacement cascades. *Science* **332**, 1196–1201 (2011).

[2] Zhang, D. Y. & Winfree, E. Control of DNA strand displacement kinetics using toehold exchange. *J. Am. Chem. Soc.* **131**, 17303–17314 (2009).

[3] Srinivas, N. *et al.* On the biophysics and kinetics of toehold-mediated DNA strand displacement. *Nucleic Acids Res.* **41**, 10641–10658 (2013).

[4] Integrated DNA Technologies. Chemical synthesis and purification of oligonucleotides. (2005).

[5] Zhang, D. Y. & Winfree, E. Robustness and modularity properties of a non-covalent DNA catalytic reaction. *Nucleic Acids Res.* **38**, 4182–4197 (2010).
